# Serum microRNA miR-501-3p as a potential biomarker related to the progression of Alzheimer’s disease

**DOI:** 10.1186/s40478-017-0414-z

**Published:** 2017-01-31

**Authors:** Norikazu Hara, Masataka Kikuchi, Akinori Miyashita, Hiroyuki Hatsuta, Yuko Saito, Kensaku Kasuga, Shigeo Murayama, Takeshi Ikeuchi, Ryozo Kuwano

**Affiliations:** 1 Department of Molecular Genetics, Bioresource Science Branch, Center for Bioresources, Brain Research Institute, Niigata University, Niigata, Japan; 2 Department of Neuropathology, Tokyo Metropolitan Geriatric Hospital and Institute of Gerontology, Tokyo, Japan; 3 Department of Pathology, National Center Hospital of Neurology and Psychiatry, Tokyo, Japan; 4 Center for Transdisciplinary Research, Niigata University, Niigata, Japan; 5 Asahigawaso Research Institute, Asahigawaso Medical-Welfare Center, Okayama, Japan; 6 Department of Molecular Genetics, Brain Research Institute, Niigata University, 1-757 Asahimachi, Niigata, 951-8585 Japan

**Keywords:** Alzheimer’s disease, Blood-based biomarker, microRNA, miR-501-3p, Next-generation sequencing, Autopsied brain, Braak staging

## Abstract

MicroRNAs (miRNAs) are attractive molecules to utilize as one of the blood-based biomarkers for neurodegenerative disorders such as Alzheimer’s disease (AD) because miRNAs are relatively stable in biofluid, including serum or plasma. To determine blood miRNA biomarkers for AD with next-generation sequencing genome-wide, we first surveyed 45 serum samples. These came from 27 AD patients and 18 controls (discovery set) that underwent autopsy within two weeks after their serum sampling and were neuropathologically diagnosed. We found that three miRNAs, hsa-miR-501-3p, hsa-let-7f-5p, and hsa-miR-26b-5p, were significantly deregulated between the AD samples and the controls. The deregulation for hsa-miR-501-3p was further confirmed by quantitative reverse transcription polymerase chain reaction (PCR) in a validation set composed of 36 clinically diagnosed AD patients and 22 age-matched cognitively normal controls with a sensitivity and specificity of 53% and 100%, respectively (area under the curve = 0.82). Serum hsa-miR-501-3p levels were downregulated in AD patients, and its lower levels significantly correlated with lower Mini-Mental State Examination scores. Contrary to its serum levels, we found that hsa-miR-501-3p was remarkably upregulated in the same donors’ AD brains obtained at autopsy from the discovery set. The hsa-miR-501-3p overexpression in cultured cells, which mimicked the hsa-miR-501-3p upregulation in the AD brains, induced significant downregulation of 128 genes that overrepresented the Gene Ontology terms, DNA replication, and the mitotic cell cycle. Our results suggest that hsa-miR-501-3p is a novel serum biomarker that presumably corresponds to pathological events occurring in AD brains.

**Electronic supplementary material:**

The online version of this article (doi:10.1186/s40478-017-0414-z) contains supplementary material, which is available to authorized users.

## Introduction

Alzheimer’s disease (AD) is the most common form of dementia in the elderly and is characterized by two neuropathological hallmarks: extracellular senile plaques (SPs), composed of β-amyloid (Aβ) peptides, and intraneuronal neurofibrillary tangles (NFTs) composed of abnormally hyperphosphorylated tau. To facilitate the clinical diagnosis of AD, numerous studies have focused on biomarkers based on neuroimaging and molecules in cerebrospinal fluid (CSF) and peripheral blood. For example, neuroimaging using positron emission tomography with the tracer carbon-11-labeled Pittsburgh compound B can detect Aβ deposits in the brains of living subjects. However, neuroimaging tests are expensive and available at only a limited number of laboratories. As a result, access to neuroimaging is confined to a small number of patients. Similarly, Aβ and tau protein levels in CSF are well-established biomarkers with high accuracy for AD [8, 15], but CSF sampling by lumbar puncture is relatively invasive and requires skilled training. In contrast, blood sampling is less invasive and available at routine examinations. Once useful blood-based biomarkers are established, they allow physicians to screen a larger number of subjects. Recent studies have reported potential blood-based biomarkers for AD from various molecular species, such as protein, lipid, and nucleic acid [23].

MicroRNA (miRNA) is considered one of the candidates for blood-based biomarkers. miRNAs are ~22 nt small noncoding RNAs that bind to the 3′ untranslated region of their target mRNAs to direct posttranscriptional repression of the target genes by forming the RNA-induced silencing complex, which leads to mRNA destabilization or translational inhibition [5, 6]. Some miRNAs are encapsulated in microvesicles, such as exosomes, and present as a relatively stable form in biofluid, including serum or plasma [39]. Recently, some sets of blood miRNAs have been shown to differentiate AD or mild cognitive impairment (MCI) from healthy controls [12, 21, 28, 32, 36, 42, 45]. For example, plasma levels of the miR-132 family (miR-128, miR-132, and miR-874) have distinguished MCI from age-matched controls with high accuracy (84–94% sensitivity and 96–98% specificity) [42]. In addition, alterations of miRNAs have also been observed in AD brains [20, 22, 41]. Hébert et al. have shown that in AD brains the miR-29a/b-1 cluster levels are decreased and correlated with the high BACE1 expression that leads to Aβ generation, suggesting that these miRNAs directly regulate the *BACE1* gene expression [22]. Despite the increasing evidence of miRNAs for AD pathology, their relationship between peripheral blood levels and brain levels, especially in the same individuals, is largely unexplored.

Here, we identified hsa-miR-501-3p as a novel serum miRNA biomarker that was downregulated in AD patients. We measured hsa-miR-501-3p levels in the same donors’ brains that were obtained at autopsy within two weeks after their serum sampling and found remarkable upregulation of hsa-miR-501-3p. To elucidate the effects of the hsa-miR-501-3p upregulation in the AD brains, we performed hsa-miR-501-3p overexpression analysis in cultured cells.

## Materials and methods

### Subjects

We prepared two independent sample sets to discover and validate potential serum miRNA biomarkers for AD. The discovery set, ROW, consisted of samples obtained from the Japanese Brain Bank Network for Neuroscience Research (JBBNNR), where all autopsied brains are systematically diagnosed with Braak staging [10, 11] and stored at −80 °C. We used serum that was collected from donors in routine blood tests within two weeks before their death. The temporal cortex tissues from the same donors were dissected by experts at JBBNNR and stored at −80 °C until use.

The validation set, NIG, was composed of clinically diagnosed subjects from Niigata University Medical and Dental Hospital. The diagnosis of AD was made by the criteria of the National Institute of Neurological and Communicative Diseases and the Stroke–Alzheimer’s Disease and Related Disorders Association [38]. Serum was collected from the subjects during the clinical follow-up period in the outpatient department and stored at −80 °C until analysis.

### *APOE* genotyping

Isolation of genomic DNA from brain or blood samples and *APOE* genotyping were performed as previously described [29].

### RNA extraction

Cell-free RNA was isolated from 200 μl of serum with miRNeasy Serum/Plasma Kit (QIAGEN, Hilden, Germany). For quantitative reverse transcription PCR (qRT-PCR) assay of the NIG validation set, 5.6 × 10^8^ copies of synthetic cel-miR-39-3p (miRNeasy Serum/Plasma Spike-In Control; QIAGEN) were added to each serum sample to normalize and monitor extraction efficiency. Finally, serum RNA was eluted in 14 μl of RNase-free water.

Total RNA was isolated from a small piece of the frozen temporal cortex tissue or cultured cells with TRIzol Plus RNA Purification Kit (Thermo Fisher Scientific, Waltham, MA, USA). The isolated total RNA was loaded on the Agilent 2100 Bioanalyzer instrument (Agilent Technologies, Santa Clara, CA, USA) to determine the value of the RNA integrity number (RIN), which indicates the quality of mRNA transcripts and ranges from 1 (totally degraded) to 10 (intact).

### Small RNA sequencing

We prepared small RNA sequencing libraries with the TruSeq Small RNA Sample Prep Kit (Illumina, San Diego, CA, USA), using 1 μg of total RNA or 5 μl of the 14 μl purified serum RNA as input. Each library was sequenced on Illumina Genome Analyzer IIx (GAIIx), and more than 20,000,000 single-end reads were obtained from each library. Adapter sequences were trimmed from the sequenced reads with cutadapt [37], and then the reads were mapped using bowtie [30] against the human genome build hg19. The number of mapped reads was counted using HTSeq [3] in each miRNA from miRBase release 20 [26]. The raw read counts were normalized by the median-of-ratios method implemented in DESeq2 [35]. To remove low-abundance miRNAs, we filtered out miRNAs with <50 mean normalized read counts across all samples. We used DESeq2 [35] to identify deregulated miRNAs between AD and controls. We also performed differential miRNA expression analysis with DESeq2’s design formula including the additional covariates, age, sex, *APOE* genotype, and hemolysis ratio or RIN, to adjust for these variables. *P*-values were calculated by the Wald test and adjusted by the Benjamini–Hochberg procedure [7] for multiple testing correction. We considered miRNAs with <0.05 adjusted *p*-value (5% false discovery rate (FDR)) statistically significant.

### Evaluation of hemolysis

To detect the degree of hemolysis during serum preparation, free hemoglobin levels were measured spectrophotometrically. 12 μl of serum was diluted with 48 μl of phosphate buffered saline and then used to determine the ratio of the absorbance at 414 nm and 375 nm, hemolysis ratio (*A*_414/375_), whose higher values show hemolyzed samples [25, 49]. We removed serum samples with >2 hemolysis ratio from the NIG validation set.

### qRT-PCR

Candidate serum miRNAs were validated by qRT-PCR using TaqMan MicroRNA Assays (Thermo Fisher Scientific). 5 μl of serum RNA was reverse transcribed to cDNA with the TaqMan MicroRNA Reverse Transcription kit using an RT primer pool containing three target miRNA primers (hsa-miR-501-3p, hsa-let-7f-5p, and hsa-miR-26b-5p) and two normalization miRNA primers (cel-miR-39-3p and hsa-miR-451a) at a final concentration of 0.2 × each in a reaction volume of 15 μl. 5 μl of the RT product was preamplified for 12 cycles with TaqMan PreAmp Master Mix using a preamplification primer pool containing those five miRNA primers at a final concentration of 0.03 × each in a reaction volume of 25 μl. The preamplified product was diluted by adding 100 μl of 0.1 × TE. Using 5 μl of the diluted product, qPCR was performed in triplicate in a reaction volume of 20 μl in a 384-well plate on an ABI PRISM 7900HT instrument with TaqMan MicroRNA Assays for each miRNA. Relative expression levels of miRNAs were calculated by the 2^–ΔΔ*C*^_T_ method [34] using cel-miR-39-3p (spike-in) and hsa-miR-451a (endogenous control) for normalization.

### Cell culture and miRNA transfection

A human neuroblastoma cell line of SH-SY5Y cells was cultured in a 1:1 mixture of minimum essential medium and Ham’s F-12 nutrient mixture supplemented with 10% fetal bovine serum and 1% penicillin–streptomycin in a CO_2_ incubator. One day after seeding, the cells were transfected with either hsa-miR-501-3p mimic (MC12927; Thermo Fisher Scientific) or scramble control (mirVana™ miRNA Mimic, Negative Control #1; Thermo Fisher Scientific) at 50 nM final concentration using NeuroMag (OZ Biosciences, San Diego, CA, USA). After 24 h, the cells were treated with the TRIzol reagent and stored at −80 °C until RNA extraction.

### mRNA sequencing and gene ontology analysis

mRNA sequencing libraries were prepared using TruSeq RNA Sample Prep Kit v2 (Illumina) with 500 ng of total RNA as input. Each library was sequenced on GAIIx, and approximately 40,000,000 of 75 bp single-end reads were obtained from each library. The sequenced reads were mapped using STAR [18] against the human genome (hg19). We used DESeq2 [35] to perform differential gene expression analysis and considered genes with <0.05 adjusted *p*-value (5% FDR) statistically significant. We used DAVID 6.8 [16, 17] to perform Gene Ontology enrichment analysis on significantly differentially expressed genes, focusing on Gene Ontology category GOTERM_BP_DIRECT. We set the statistical significance threshold at 0.05 (5% FDR).

## Results

### Discovery of candidate serum miRNA biomarkers for AD

To prepare the ROW discovery set, we deliberately selected cases whose autopsied brain and serum samples alike were archived in JBBNNR. According to Braak staging [10, 11], we defined cases with Braak NFT stages IV through VI and Braak amyloid stage C as AD, and cases with Braak NFT stages 0 through II and Braak amyloid stage 0 or A as controls (Additional file 1: Figure S1) [40]. These definitions of AD and controls prevented the study sample from including transient forms of the disease (e.g., cases with Braak NFT stage III) so that we could distinctly detect the difference between patients with typical AD and controls without obvious pathological findings. As a result, 27 neuropathologically diagnosed AD patients and 18 control subjects were selected. The ROW discovery set had significant differences (*P* < 0.05) in age at death, sex, brain weight, and RIN between AD and controls (Table 1).

**Table 1:** Demographics of the two sample sets in this study

	CT	AD	*P*-value
ROW discovery set			
N	18	27	-
AAD, yr	76.3 ± 7.1	84.5 ± 8.0	0.001^a^
Gender, % (F : M)	27.8 : 72.2	63.0 : 37.0	0.033^b^
PMI, hr	9.6 ± 9.4	13.4 ± 11.6	0.158^a^
BW, g	1268 ± 127	1156 ± 120	0.003^a^
RIN			
TC	7.9 ± 0.8	7.1 ± 1.2	0.006^a^
Hemolysis ratio			
Serum	1.9 ± 0.6	1.7 ± 0.5	0.105^a^
*APOE*			
Genotype, % (ε3*3 : ε3*4 : ε4*4)	55.6 : 44.4 : 0.0	44.4 : 25.9 : 29.6	0.550^c^
Allele, % (ε3 : ε4)	77.8 : 22.2	57.4 : 42.6	0.069^c^
NIG validation set			
N	22	36	-
AAE, yr	73.7 ± 8.4	74.7 ± 7.3	0.556^a^
Gender, % (F : M)	80.0 : 20.0	63.9 : 36.1	0.333^b^
MMSE	29.3 ± 0.7	19.3 ± 5.4	4.8.E-08^a^
Hemolysis ratio			
Serum	1.3 ± 0.2	1.3 ± 0.2	0.316^a^
*APOE*			
Genotype, % (ε3*3 : ε3*4)	85.7 : 14.3	55.6 : 44.4	0.056^c^
Allele, % (ε3 : ε4)	92.9 : 7.1	77.8 : 22.2	0.090^c^

*Abbreviations*: *AAD* age at death, *AAE* age at examination, *AD* Alzheimer’s disease, *APOE* apolipoprotein E, *BW* brain weight, *CT* control, *F* female, *g* grams, *hr* hours, *M* male, *MMSE* mini-mental state examination, *PMI* postmortem interval, *RIN* RNA integrity number, *TC* temporal cortex, *yr* years

^a^Calculated by Mann-Whitney *U*-test between AD and CT

^b^Calculated by Fisher’s exact test for gender distribution

^c^Calculated by Fisher’s exact test for *APOE* ε4 allele carrier status (ε4 carrier and ε4 non-carrier)

Data are presented as the mean ± standard deviation

**Fig. 1:**
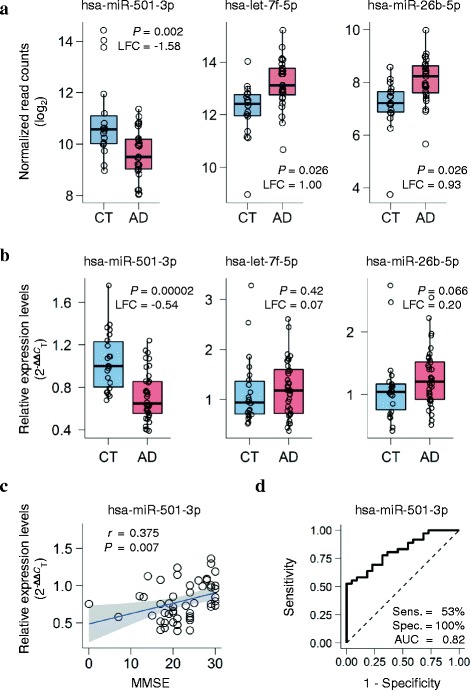
A novel serum miRNA biomarker hsa-miR-501-3p that differentiates patients with Alzheimer’s disease (AD) from controls (CT). **a** Serum miRNA levels of the three candidates significantly deregulated between AD and controls in the ROW discovery set. Normalized read counts from next-generation sequencing data were converted to a log2 scale and plotted against the disease status. The lower hinge, the line in the middle, and the upper hinge of the box plot show the 25th percentile, 50th percentile (median), and 75th percentile, respectively. The lower whisker extends from the lower hinge to the lowest value that is within 1.5 times the interquartile range (IQR) of the hinge. The upper whisker extends from the upper hinge to the highest value that is within 1.5 times the IQR. LFC: log2 fold change; *P*: *p*-value adjusted by the Benjamini–Hochberg procedure for multiple testing correction. **b** Three candidates’ serum miRNA levels quantified by quantitative reverse transcription PCR (qRT-PCR) in the NIG validation set. Relative expression levels of the miRNAs were calculated using the 2^–ΔΔ*C*^_T_ method with cel-miR-39-3p (spike-in) and hsa-miR-451a (endogenous control) for normalization and were plotted against the disease status. *P*-values were computed using the Mann–Whitney *U*-test between AD and controls. Box plots display the distributions of data in the same way as in Fig. 1a. LFC: log2 fold change. **c** Significant positive correlation between Mini-Mental State Examination (MMSE) scores and serum hsa-miR-501-3p levels in the NIG validation set. Relative expression levels from the qRT-PCR data were plotted against MMSE. *r* shows the Spearman rank correlation coefficient. The blue line shows a linear regression line, and a shaded gray area around the line represents 95% confidence intervals. **d** The receiver-operating characteristic (ROC) curve of serum hsa-miR-501-3p in the NIG validation set. The ROC curve analysis showed 53% sensitivity and 100% specificity. The area under the ROC curve (AUC) was 0.82

We isolated cell-free RNA from serum collected from the donors within two weeks before their death. The serum RNA was analyzed by high-throughput next-generation sequencing (NGS) to compare miRNA levels between AD and controls. We filtered out low-abundance miRNAs by mean normalized read counts. Of the remaining 148 miRNAs, 3 miRNAs had significant changes in expression level: hsa-miR-501-3p (adjusted *P* = 0.002, log2 fold change = −1.58), hsa-let-7f-5p (adjusted *P* = 0.026, log2 fold change = 1.00), and hsa-miR-26b-5p (adjusted *P* = 0.026, log2 fold change = 0.93). In the AD patients, hsa-miR-501-3p was downregulated and the other two were upregulated (Fig. 1a). In particular, hsa-miR-501-3p still had a significant change in expression level after adjusting for age, sex, *APOE* genotype, and hemolysis ratio (adjusted *P* = 0.004, log2 fold change = −2.07; Additional file 2: Table S1). We added the hemolysis ratio of serum samples to those covariates because hemolysis could affect some miRNAs’ levels [25].

### Validation of candidate serum miRNAs

To validate the three candidate serum miRNAs, we prepared sample set NIG, which was composed of 36 clinically diagnosed AD patients and 22 age-matched cognitively normal controls (Table 1). Serum was collected from those subjects and examined for the degree of hemolysis in the same way as the ROW discovery set. To evaluate more strictly the miRNA levels, we excluded the serum samples with a >2 hemolysis ratio from the NIG validation set according to Zanutto et al. [49]. Cell-free RNA was isolated from the nonhemolyzed serum samples and analyzed by qRT-PCR to quantify the miRNA levels. The qRT-PCR analysis revealed that hsa-miR-501-3p had a significant, consistent change in expression level (*P* = 0.00002, log2 fold change = −0.54; Fig. 1b), whereas hsa-miR-26b-5p showed marginal significance (*P* = 0.066, log2 fold change = 0.20; Fig. 1b). Furthermore, serum hsa-miR-501-3p levels significantly correlated with Mini-Mental State Examination scores (*r* = 0.375, *P* = 0.007; Fig. 1c). The receiver-operating characteristic curve of hsa-miR-501-3p showed 53% sensitivity and 100% specificity (area under the curve = 0.82; Fig. 1d). These results suggest that hsa-miR-501-3p could be a novel serum biomarker for clinical diagnosis of AD.

### Deregulated hsa-miR-501-3p in AD brains

**Fig. 2:**
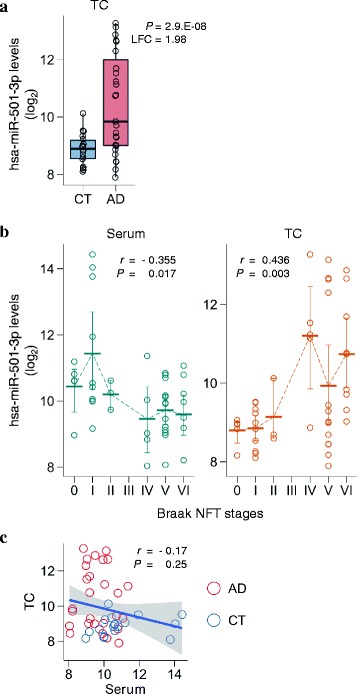
Deregulated hsa-miR-501-3p not only in the serum but also in the brain of the same individuals over the course of Alzheimer’s disease (AD) progression. **a** Brain hsa-miR-501-3p levels prominently upregulated in the AD brains of the ROW discovery set. The levels of hsa-miR-501-3p in the temporal cortex were analyzed using next-generation sequencing (NGS). Normalized read counts from the NGS data were converted to a log2 scale and plotted against the disease status. Box plots display the distributions of data in the same way as in Fig. 1a. CT: control; LFC: log2 fold change; *P*: *p*-value adjusted by the Benjamini–Hochberg procedure for multiple testing correction; TC: temporal cortex. **b** Significant correlation of either serum hsa-miR-501-3p levels or brain hsa-miR-501-3p levels with Braak NFT stages in the ROW discovery set. While serum hsa-miR-501-3p levels negatively correlated with Braak NFT stages (Spearman’s *r* = −0.355, *P* = 0.017), brain hsa-miR-501-3p levels positively correlated with Braak NFT stages (Spearman’s *r* = 0.436, *P* = 0.003). Normalized read counts from the NGS data were converted to a log2 scale and plotted against Braak NFT stages. Each thick bar shows the mean value of hsa-miR-501-3p levels in a Braak NFT stage. Each error bar shows a 95% confidence interval of hsa-miR-501-3p levels in a Braak NFT stage. TC: temporal cortex. **c** Negative correlation between serum hsa-miR-501-3p levels and brain hsa-miR-501-3p levels in the same individuals of the ROW discovery set. The levels of hsa-miR-501-3p between serum and brain of the same individuals were insignificantly yet negatively correlated (Spearman’s *r* = −0.17, *P* = 0.25). Normalized read counts of hsa-miR-501-3p levels were converted to a log2 scale and plotted. The *x*-axis and *y*-axis show serum hsa-miR-501-3p levels and brain hsa-miR-501-3p levels, respectively. The blue line shows a linear regression line, and a shaded gray area around the line represents 95% confidence intervals. Red circles and blue circles show the hsa-miR-501-3p levels of AD patients and control subjects, respectively. CT: control; TC: temporal cortex

To investigate whether serum hsa-miR-501-3p levels had an association with brain levels, we measured hsa-miR-501-3p levels in the same donors’ brains that were obtained at autopsy within two weeks after their serum sampling. We used those autopsied brains in the ROW discovery set and isolated total RNA from their temporal cortex, a brain region that is severely affected in the late stages of AD progression [11]. The total RNA was analyzed by NGS to compare miRNA levels between AD and controls. The NGS analysis revealed that 187 of 472 miRNAs were significantly deregulated (adjusted *P* < 0.05; Additional file 2: Table S2). This included hsa-miR-501-3p, which was prominently upregulated in the AD brains and had the third lowest *p*-value (adjusted *P* = 2.9.E–08, log2 fold change = 1.98, rank = 3; Fig. 2a and Additional file 2: Table S2). We found that hsa-miR-501-3p still had a significant change in expression level after adjusting for age, sex, *APOE* genotype, and RIN (adjusted *P* = 0.0008, log2 fold change = 1.33, rank = 25; Additional file 2: Table S3). Moreover, hsa-miR-501-3p levels significantly correlated with Braak NFT stages (Fig. 2b). As AD pathology progressed, brain hsa-miR-501-3p levels increased (*r* = 0.436, *P* = 0.003) while its serum levels decreased (*r* = −0.355, *P* = 0.017). We also observed the negative correlation between brain hsa-miR-501-3p levels and serum hsa-miR-501-3p levels in the same individuals, although it was not significant (*r* = −0.17, *P* = 0.25; Fig. 2c). Collectively, hsa-miR-501-3p was deregulated in the brain as well as in the serum of the same individuals during AD progression.

### Functional assays of hsa-miR-501-3p in cultured cells

To elucidate the effects of the hsa-miR-501-3p upregulation in AD brains, we transfected synthetic hsa-miR-501-3p into SH-SY5Y cells. Total RNA isolated from the cultured cells was analyzed by NGS to identify genes that are regulated by hsa-miR-501-3p. Overexpression of hsa-miR-501-3p in SH-SY5Y cells resulted in significant changes in the expression levels of 208 genes in comparison to the scramble control (adjusted *P* < 0.05; Additional file 2: Table S4). Of the 208 significant genes, 128 were downregulated in hsa-miR-501-3p overexpression. To investigate whether hsa-miR-501-3p directly represses the gene expression of those downregulated genes we used TargetScan 7.1, a database of predictive targets of each miRNA [2]. Of the 128 downregulated genes, 123 were annotated by TargetScan 7.1, which predicted that hsa-miR-501-3p bound to 71 of the 123 genes (71/123 (58%)). This rate of predictive targets of hsa-miR-501-3p was significantly higher than that of all the genes tested and annotated by TargetScan 7.1 (1788/11483 (16%), Fisher’s exact test, *P* < 9.7.E–16), supporting that hsa-miR-501-3p overexpression successfully induced alteration of the gene expression in the cultured cells. To uncover the biological function of the genes that were altered in hsa-miR-501-3p overexpression, we used DAVID 6.8 [16, 17] to perform Gene Ontology enrichment analysis on the 208 significant genes. DAVID 6.8 revealed that the set of the 128 downregulated genes significantly overrepresented some terms of Gene Ontology, such as DNA replication and mitotic cell cycle (Additional file 2: Table S5). No significant enrichment was observed in the remaining 80 upregulated genes (Additional file 2: Table S6).

## Discussion

There is a significant link between the developments of SPs and NFTs in the brain and the alterations of Aβ and tau protein levels in CSF, thus making CSF levels useful biomarkers that facilitate clinical diagnosis of AD. In this study, we found that serum hsa-miR-501-3p levels were downregulated while brain levels were prominently upregulated in the AD patients of the ROW discovery set. Both serum and brain hsa-miR-501-3p levels significantly correlated with Braak NFT stages, suggesting that those hsa-miR-501-3p levels are related to the disease progression. In addition, the alteration of serum hsa-miR-501-3p was replicated in the NIG validation set composed of clinical samples. These results indicate that serum hsa-miR-501-3p is a plausible biomarker for AD and its serum levels presumably correspond to its brain levels, which links to AD pathology. There was up to a two-week time lag between the serum sampling and brain autopsy in the ROW discovery set, but a time of two weeks lends little difference according to the gradual progression of AD pathology over years. Therefore, we consider our serum and brain samples in the ROW discovery set to be collected from each donor at approximately the same time point.

To our knowledge, alteration of hsa-miR-501-3p in peripheral blood has never been reported in preceding AD biomarker studies. This is probably due to technical differences, including different methods of RNA extraction [14, 33], different quantitative techniques [47], and different sources as input such as plasma, serum, and whole blood. For instance, miRNA contents from blood cells are much greater than those from cell-free plasma or serum [47]. Hence, whole blood samples or highly hemolyzed samples including contaminants of blood cells may result in outcomes different from serum or plasma samples. In fact, we discovered hsa-let-7f-5p and hsa-miR-26b-5p that were upregulated in serum, whereas Leidinger et al. [32] have reported decreased levels in whole blood from AD patients. The different starting materials, namely serum or whole blood, may lead to the inconsistent results. Consequently, it is vital to follow a standardized protocol stringently to achieve a consensus on miRNA biomarkers.

Few studies have described the deregulation of hsa-miR-501-3p in AD brains as well as peripheral blood. This may be attributed to the difference in Braak NFT stages of autopsied brain samples. Our NGS analysis in the ROW discovery set showed that brain hsa-miR-501-3p levels did not continuously rise over the Braak NFT stages; its levels temporarily dropped in Braak NFT stage V (Fig. 2b). Thus, which Braak NFT stages researchers define for AD and controls can determine the outcome of a study. However, the NGS analysis revealed the deregulation of miRNAs that other studies have described (e.g., miR-107 [46], miR-125b [4], and the miR-132/212 cluster [31, 43, 48]). As Lau et al. have shown [31], further work is needed to uncover the pattern of miRNA levels over the Braak NFT stages with dozens of brain samples from different cortical regions in each Braak NFT stage.

Although it remains elusive as to how the upregulation of hsa-miR-501-3p in the brain has an effect on AD pathogenesis, our results indicate a possibility that hsa-miR-501-3p upregulation could cause alterations in the cell cycle in AD brains. We found that hsa-miR-501-3p overexpression in cultured cells induced the downregulation of 128 genes that were significantly enriched in the biological processes of DNA replication and mitotic cell cycle (Additional file 2: Table S5). Accumulating evidence indicates that inappropriate cell cycle reentry in postmitotic neurons, which leads to apoptotic cell death, is an early sign that precedes the developments of SPs and NFTs in AD brains [1, 9, 13, 19, 27, 44]. Alternatively, Hu et al. [24] have recently demonstrated in rat brains that miR-501-3p mediates the activity-dependent regulation of the expression of the AMPA receptor subunit GluA1 in dendrites, suggesting that miR-501-3p contributes to synaptic plasticity related to cognitive functions, including learning and memory.

## Conclusions

In summary, we discovered that hsa-miR-501-3p is strongly suggested as a novel serum biomarker for AD. Our study associated the downregulation of serum hsa-miR-501-3p levels with its remarkable upregulation in AD brains, which may be involved in the pathogenesis of AD through an aberrant neuronal cell cycle. Our study suggests that serum hsa-miR-501-3p is a novel indicator that presumably reflects the progression of AD.

## Additional files

Additional file 1: Figure S1. This study’s definitions of patients with Alzheimer’s disease (AD) and controls on the basis of Braak staging in the ROW discovery set. (PDF 186 kb)
Additional file 2: Table S1. Top 20 deregulated serum miRNAs that were identified by NGS in the ROW discovery set after adjusting for age, sex, *APOE* genotype, and hemolysis ratio. Table S2. Significantly deregulated miRNAs that were identified by NGS in the temporal cortex of the ROW discovery set. Table S3. Significantly deregulated miRNAs that were identified by NGS in the temporal cortex of the ROW discovery set after adjusting for age, sex, *APOE* genotype, and RIN. Table S4. Significantly differentially expressed genes that were identified by NGS in hsa-miR-501-3p overexpression in cultured cells. Table S5. Gene Ontology enrichment analysis on the significantly downregulated genes in hsa-miR-501-3p overexpression in cultured cells. Table S6. Gene Ontology enrichment analysis on the significantly upregulated genes in hsa-miR-501-3p overexpression in cultured cells. (XLS 172 kb)

## Abbreviations

AD: Alzheimer’s disease
Aβ: β-amyloid
CSF: Cerebrospinal fluid
FDR: False discovery rate
GAIIx: Genome Analyzer IIx
JBBNNR: Japanese Brain Bank Network for Neuroscience Research
MCI: Mild cognitive impairment
miRNA: microRNA
NFT: Neurofibrillary tangle
NGS: Next-generation sequencing
qRT-PCR: Quantitative reverse transcription PCR
RIN: RNA integrity number
SP: Senile plaque
