# Supplementary material for: Serum microRNA miR-501-3p as a potential biomarker related to the progression of Alzheimer’s disease
Source: Acta Neuropathol Commun. 2017 Jan 31;5:10. doi: 10.1186/s40478-017-0414-z (PMC5282710; doi:10.1186/s40478-017-0414-z)
Supplement: Additional file 1: Figure S1. — This study’s definitions of patients with Alzheimer’s disease (AD) and controls on the basis of Braak staging in the ROW discovery set. (PDF 186 kb) [file 40478_2017_414_MOESM1_ESM.pdf]

Figure S1

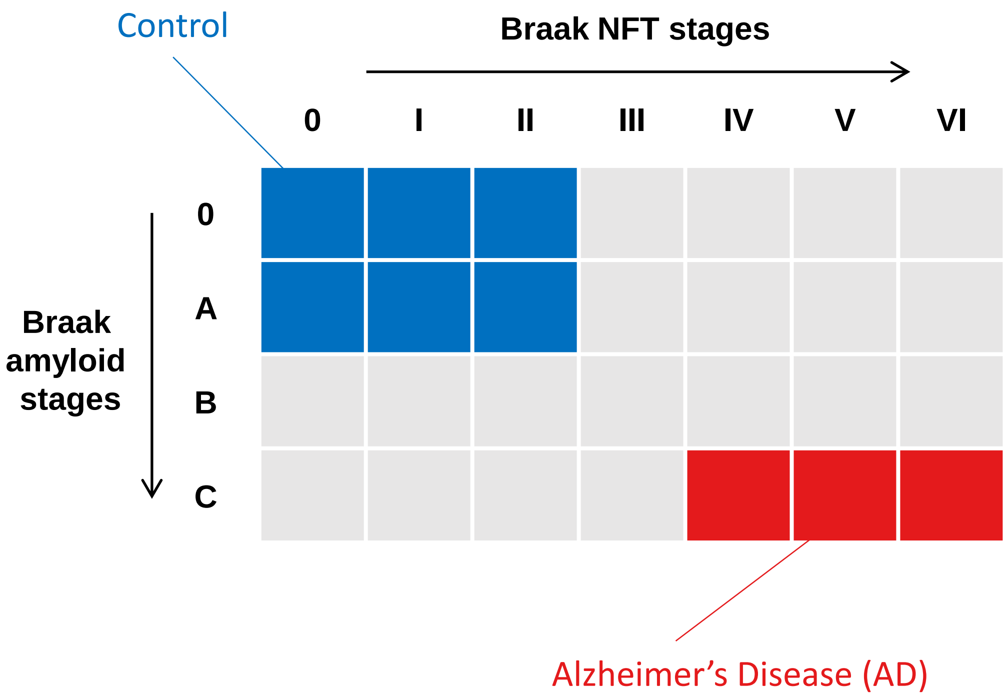

Figure S1. This study's definitions of patients with Alzheimer's disease (AD) and controls on the basis of Braak staging in the ROW discovery set. We defined cases with Braak NFT stage IV through VI and Braak amyloid stage C as AD (red-colored cells), and cases with Braak NFT stage 0 through II and Braak amyloid stage 0 or A as controls (blue-colored cells).
